# Successes and challenges of health systems governance towards universal health coverage and global health security: a narrative review and synthesis of the literature

**DOI:** 10.1186/s12961-022-00858-7

**Published:** 2022-05-02

**Authors:** Ayal Debie, Resham B. Khatri, Yibeltal Assefa

**Affiliations:** 1 Department of Health Systems and Policy, Institute of Public Health, College of Medicine and Health Sciences, University of Gondar, P.O. Box 196, Gondar, Ethiopia; 2 School of Public Health, The University of Queensland, Brisbane, Australia

**Keywords:** Health systems governance, Universal health coverage, Global health security

## Abstract

**Background:**

The shift in the global burden of disease from communicable to noncommunicable was a factor in mobilizing support for a broader post-Millennium Development Goals (MDGs) health agenda. To curb these and other global health problems, 193 Member States of the United Nations (UN) became signatories of the Sustainable Development Goals (SDGs) and committed to achieving universal health coverage (UHC) by 2030. In the context of the coronavirus disease 2019 (COVID-19) pandemic, the importance of health systems governance (HSG) is felt now more than ever for addressing the pandemic and continuing to provide essential health services. However, little is known about the successes and challenges of HSG with respect to UHC and health security. This study, therefore, aims to synthesize the evidence and identify successes and challenges of HSG towards UHC and health security.

**Methods:**

We conducted a structured narrative review of studies published through 28 July 2021. We searched the existing literature using three databases: PubMed, Scopus and Web of Science. Search terms included three themes: HSG, UHC and health security. We synthesized the findings using the five core functions of HSG: policy formulation and strategic plans; intelligence; regulation; collaboration and coalition; and accountability.

**Results:**

A total of 58 articles were included in the final review. We identified that context-specific health policy and health financing modalities helped to speed up the progress towards UHC and health security. Robust health intelligence, intersectoral collaboration and coalition were also essential to combat the pandemic and ensure the delivery of essential health services. On the contrary, execution of a one-size-fits-all HSG approach, lack of healthcare funding, corruption, inadequate health workforce, and weak regulatory and health government policies were major challenges to achieving UHC and health security.

**Conclusions:**

Countries, individually and collectively, need strong HSG to speed up the progress towards UHC and health security. Decentralization of health services to grass root levels, support of stakeholders, fair contribution and distribution of resources are essential to support the implementation of programmes towards UHC and health security. It is also vital to ensure independent regulatory accreditation of organizations in the health system and to integrate quality- and equity-related health service indicators into the national social protection monitoring and evaluation system; these will speed up the progress towards UHC and health security.

**Supplementary Information:**

The online version contains supplementary material available at 10.1186/s12961-022-00858-7.

## Background

The impact of the Millennium Development Goals (MDGs) on the progress towards global health goals was impressive; however, the progress was uneven [1]. The shift in the global burden of disease from communicable to noncommunicable was also a factor in mobilizing support for a broader post-MDG health agenda [1]. In 2015, the 193 Member States of the United Nations became signatories of the Sustainable Development Goals (SDGs) [2] and committed to achieving health-related Sustainable Development Goal 3 (SDG3 and universal health coverage (UHC) by 2030. Achieving UHC is the global slogan for all strata of populations within countries to gain the right to health [3, 4].

UHC is a system whereby all people and communities have access to quality health services without the risk of financial hardship [11]. Health security also involves the prevention, detection and response to naturally emerging, accidental and deliberate biological threats [7, 8], focusing on preventing communicable diseases, natural and human-made disasters, conflicts and other emergencies [12]. The interconnection of UHC and health security are two sides of one coin and mutually inclusive, and cannot be achieved without common action [13]. Moving closer to UHC requires the availability of quality and affordable health services with other building blocks of the health system, including infrastructure, medicines and medical products, health workers, health information and health systems financing [14]. Countries moving towards UHC will have ripple effects in the progress of other targets of SDGs. For instance, good health allows children to learn and adults to earn, helps people escape from poverty, addresses social and gender inequities, social cohesion and health security [11]. The Astana Declaration and the SDG3 Global Action Plan sounded a wake-up call for the global health community to reimagine the health system and realign current needs to ensure suitability and rebuild resilience [15]. Health systems governance (HSG) builds solid partnerships and creates accountability mechanisms to respond to health emergencies and maintain health security [16].

Good HSG is key to achieving UHC and health security [5]. The application of strong HSG has assisted in attaining UHC [6] and health security from naturally emerging, accidental and biological threats [7, 8]. However, the realization of UHC and health security requires strong leadership and political support to strengthen health institutions for successful implementation [9, 10].

HSG involves the actions and means adopted by society to organize to promote and protect the health of its population [17, 18]. Governance involves policy guidance, coordination, regulation and accountability to ensure equity, efficiency and sustainability [19]. The core functions of HSG, based on the WHO stewardship framework, include the following: policy formulation and strategic plans; generate intelligence; design of regulation; collaboration and coalition; and ensuring accountability [14]. In addition, HSG involves a wide range of steering and rule-making functions to ensure UHC [20] and attain health security [7, 8].

The COVID-19 pandemic also demonstrates that effective governance is crucial for health security [21]. The COVID-19 pandemic disproportionately affects poorer communities and socially excluded people. Exclusion from work due to health problems can easily result in economic impoverishment and inequitable healthcare access, which will undoubtedly worsen health status [22]. The previous body of literature has mainly focused on HSG concerning health services delivery; however, it is still lacking in the systematic mapping of evidence on driving factors in effective HSG that can have implications to achieve UHC and health security. This review, therefore, aims to assess the successes and challenges of HSG towards UHC and health security. This review helps countries and the global health community, including policy-makers and health programmers, to improve HSG by informing the revision of their plans, policies and strategies.

## Methods

### Design

A structured narrative review was conducted through reviewing studies published until 28 July 2021 using the five WHO core functions of HSG stewardship framework components [14].

### Search strategy

Three databases (PubMed, Scopus and Web of Science) were used to identify all published articles. Google and Google Scholar were also used to search additional literature on 28 July 2021. The search terms included three themes: HSG, UHC and health security. Several search terms were identified under those three themes (Additional file 1), and the search strategies built using “Title/Abstract” by linking “AND” and “OR” Boolean operator terms as appropriate. In addition, we used the Enhancing Transparency in Reporting the Synthesis of Qualitative Research (ENTREQ) checklist for reporting the articles (Additional file 2). We also followed some components of the Preferred Reporting Items for Systematic Reviews and Meta-Analyses (PRISMA) guidelines to present this paper as a narrative review.

### Inclusion and exclusion criteria

All retrieved studies were initially imported into the Endnote library to assist in removing the duplicates. After removing the duplicates, two authors (AD and RBK) independently screened the articles by title and abstract based on eligibility criteria. The quality of all eligible retrieved articles was assessed by the three independent reviewers using the Joanna Briggs Institute's (JBI) critical appraisal checklist for qualitative research. The senior author (YA) mediated through discussion with the reviewers when there were any discrepancies between them. We retained the full texts of all relevant studies found to meet the inclusion criteria for the final synthesis (Table 1).

**Table 1:** Eligibility criteria of articles/books on HSG towards UHC and health security, 2021

Characteristics	Inclusion criteria	Exclusion criteria
Publication time	All published articles until 28 July 2021 were included	Articles published after 28 July 2021 were excluded
Language	Articles published in English were included	Articles published in languages other than English were excluded
Types of articles	All articles on HSG towards UHC and global health security were included irrespective of the type of article and methodology	Articles which did not identify the successes or challenges of HSG towards UHC and health security were excluded

### Data extraction and synthesis

We used Microsoft Excel for data extraction. The template contained the name of the first author, year of publication, title, setting/ location, design, type of article, data sources, methods of analysis and key outcomes. We only extracted qualitative information from all eligible articles, including the quantitative studies. Data extraction was conducted by the two authors (AD and RBK) independently to reduce risk of bias. We also conducted a double check-up and verification of the extracted information. The applicability of this review included studies that assessed HSG to ensure its impact on the attainment of UHC and health security. Governance in the health sector is a wide range of steering and rule-making functions carried out by decision-makers to achieve national health policy objectives [20]. The core functions of governance mainly comprise policy formulation and strategic plans; generate intelligence; design of regulation; building collaboration and coalition; and ensure accountability [14], which are the attributing factors for attaining UHC and health security.

### Framework for data synthesis

The phenomena of interest in this review were the application of strong HSG for the attainment of UHC, which includes equity and quality of health services and protection of financial risk [6], and health security comprising the prevention, detection and response to naturally emerging, accidental and deliberate biological threats [7, 8]. HSG includes the actions and means adopted by society to organize to promote and protect the health of its population [17, 18]. The authors initially discussed the analytical themes/framework independently and then collectively to minimize bias. The senior author (YA) cross-validated and resolved any discrepancies. An explanation of the core functions is provided in Table 2 [14].

**Table 2:** Explanations for core governance functions, 2021

Governance function	Explanation
Policy formulation and strategic plans	Development, implementation and review of national health policies, strategies and plans; national governance strategies and plans
Generate intelligence	It is the interpretation, analysis, processing and generation of useful information for decision-making and better achievement of goals. It includes generating and using data on financial catastrophe and impoverishment, health budgets and expenditure, donor commitments and disbursements
Design regulation	Design of health system organizational structures and their roles, powers and responsibilities; design of regulation; standard-setting; incentives; enforcement and sanctions
Building collaboration and coalition	Working practices for a common purpose across sectors and with external partners. This covers norms and standards for developing inclusive health plans, technical and policy support, sharing experiences, and capacity-building
Ensure accountability	Putting in place governance structures, rules and processes for health sector organizations; mechanisms for independent oversight, monitoring, review and auditing; transparent availability and publication of policies, regulations, plans, reports, accounts, etc.; and openness to scrutiny by political representatives and civil society

## Results

### Description of the reviewed articles

**Fig. 1:**
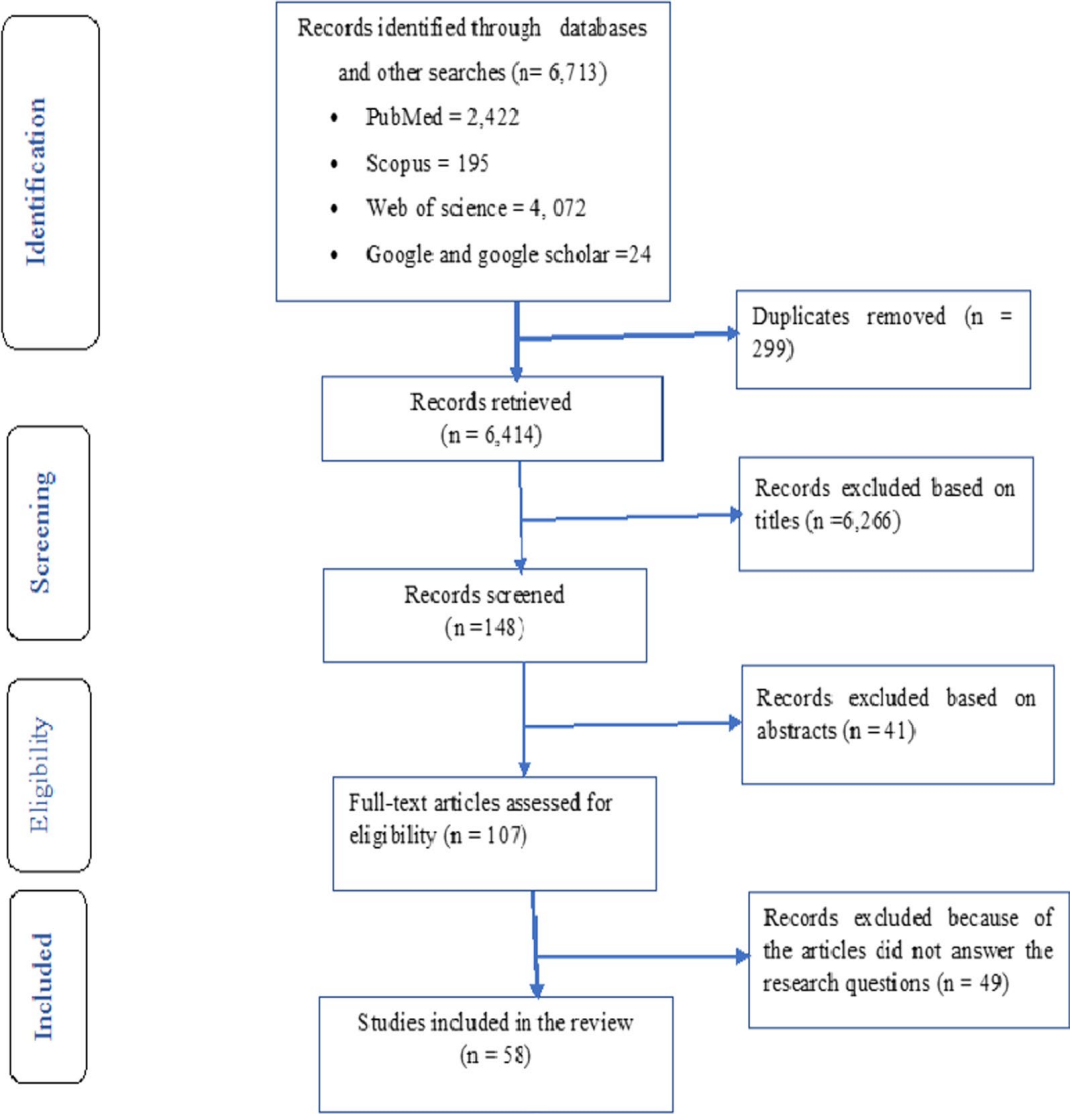
ENTREQ flow diagram for the selection of articles in the review of HSG towards UHC and health security, 2021

A total of 58 articles were included in the final review (Fig. 1). Four quantitative, eight qualitative, two mixed, 16 reviews and 28 other articles (e.g. WHO reports, books, perspectives, commentary, debate) were included for final review (Additional file 3). In addition, we included 36 articles in low- and middle-income countries (LMICs), 17 articles from the global context and four in high-income countries (HICs) in the final review (Additional file 4).

### Thematic areas for review

A framework analysis was done in this review, and the findings are presented using the five core functions of HSG. The core functions of HSG include policy formulation and strategic plans, generating intelligence, designing regulations, building collaboration and coalition, and ensuring accountability.

### Successes and challenges of HSG

We extracted data from all eligible articles related to the successes and challenges of HSG towards UHC and health security. We used the five core functions of health governance to summarize the key findings. The successes and challenges of each of the core functions of HSG towards UHC and health security are also presented separately. The health system strategies that help speed up UHC and health security are also indicated (Table 3).

**Table 3:** Successes and challenges of HSG towards UHC and health security, 2021

Framework dimension	UHC		Health security		Strategies to realize UHC and/or health security
	Successes	Challenges	Successes	Challenges	
Policy formulation and strategic plans	Single-payer scheme in Indonesia improved health equity and access after the novel UHC initiative [25] Prepaid health spending and financial risk pooling is a crucial sign of progress towards UHC [24] Public financing improves health system functioning [23] Expansion of pro-poor healthcare services resulted in good progress towards UHC in Sri Lanka [26] Decentralization of healthcare services enhances healthcare access and equity [27] Village health volunteers in Thailand, the lady health workers in Pakistan and health extension workers (HEWs) in Ethiopia are all successful community-based models which have contributed immensely towards health programmes [28] Refresher or other types of training, supervision, clear policies on reward systems and good management support helped community health workers to give good quality of care in Botswana [33]	Shortages in human resources and medical supplies; and poor physical healthcare services access [30], Poor physical access to essential health services reported in most countries in Africa [31], Disparity of health service utilization in Nigeria varies across educational level, residence, gender and socioeconomic status of the service users [29] Socially excluded population groups received health services from a dysfunctional publicly provided health system marked by gaps and often invisible barriers, which undermines the progress towards UHC[32]	Integration of human resource planning with health emergency planning assisted in controlling the cholera epidemic [34]	A centralized one-size-fits-all approach did not address the complexity and diversity in Indonesia [25] Absence of clear judicial, executive and legislative authority and clarity of structures in conflict settings, like Syria [35] Unclear administrative roles and command structures during health emergencies [16]	Resilient health workforces at country levels were critical to ensuring health security and support planning and prioritization of health issues [36] Moving away from a one-size-fits-all approach in guiding pandemic response [37] Political commitment, fair contribution and distribution of resources speed up the path of UHC [38] An economic architecture allows reducing poverty, unemployment and inequities [39] Rebuilding HSG had progressive changes on service delivery in conflict settings, like Syria [35] Conceptualization of health workforce governance enables the operationalization of governance policies to achieve UHC and global health security (GHS) [40] Effective strategic planning, regulation and management of the health workforce to combat health shocks [30] Well-designed and community-driven initiatives are a means to achieve UHC and GHS [41]
Intelligence	High-quality health information systems (HIS) enable effective monitoring of global and national health inequality [43] Community health household registers improve health system outcomes [44] Innovative data management in a unified process, assists in providing a timely response for patient care outcomes in Ethiopia [46] Mobile phones and internet are creating opportunities to improve access to appropriate knowledge and advice to realize UHC [45] Australia was the first to establish a formal national health technology assessment programme in the Asian Pacific region [42]	Neglect of effective people-centred healthcare information affected access to essential health services [47]	An independent, objective and transparent assessment of health system gaps needed to ensure early detection, prevention and response to biological threats [48] A timely response needed to meet the national and global health goals [16] Surveillance capacity and strong investments to improve the strength of the health system during crisis [16]	Late responses due to poor surveillance and lack of combining routine data weakened the functionality of plans [49]	Using indicators in the private sector and subnational levels helps ensure data quality and response to public health threats [52] Strengthening local-level managers' ability is substantial leverage in supporting informed local decision-making [51] Strategic direction to sustain the achievements of digital data culture and an automated single reporting system for multiple stakeholders to make the system user-friendly [50]
Regulation	Established national and subnational regulatory agencies are crucial to monitor and enforce laws and regulations to access emergency care [53] Centralized public regulatory authority of the redistributive funding model in the French healthcare system reduced financial barriers to access for the poor population [54] Mentorship and enhanced supervision of health staff improve quality care at health facilities [44] Regulators are encouraged to invest in gauging their performance and information sharing [60] Regulating the cost of private healthcare improves the move towards UHC [55] Close monitoring at all levels on the trends of key indicators and early corrective measures brought good health outcomes in Rwanda [56]	Political interference and unclear roles and responsibilities of different governmental regulatory bodies contributed to failures in service delivery [55] The regulatory architecture for healthcare in Mongolia resulted in poor affordability and quality of private care [55] Inadequacies of the human resource capacities of the regulatory organizations [57] Lack of enforcement for free health services to the poor in government-subsidized private hospitals in Delhi [57]	Adequate training and supportive supervision to community health workers (CHWs) are helpful to save the life of patients at the time of emergencies [58]	Inadequacies of the human resource capacities of the regulatory organizations [57]	International treaties, constitutional and statutory law, regulations, guidelines, protocols and informal practice patterns are instrumental in governing the health system and improving service delivery [59] Adopting strategic purchasing and ensuring an independent accreditation system/organization accelerates progress on healthcare quality [27] Regulation shall be exercised in medical and pharmaceutical practices in the healthcare system to improve supply chain management [60]
Collaboration and coalition	Strong partnership with national and global actors is required to avoid late responsiveness of the health system [61] Coordination with nongovernmental organizations (NGOs) and local councils in conflict settings help to address health system fragmentation [35] Coordination between public, private for-profit and not-for-profit sectors were needed to optimize the health service delivery [19] Holistic and integrated health service delivery helps to avoid resource fragmentation and improve efficiency in healthcare delivery [62] Participatory governance in health systems platforms, such as the national health assembly in Thailand, is a key pillar for achieving UHC [63]	Poor leadership practices at the subnational and national levels were the main challenges, which lead to poor coordination and absence of a prompt response to specific health shocks [64]	Multisectoral, collaborative working within and across sectors improves international health regulation (2005) [65] High-level negotiations and health diplomacy efforts in the Caribbean region resulted in the “Port of Spain Declaration” to prevent noncommunicable diseases (NCDs) [66] The Global Health Security Agenda (GHSA) speeds up the progress towards a safe and secure world from infectious disease threats [67] Collective action by all to mitigate, prevent and fight against health security threats [68]	Poor leadership practices were the main challenge for the poor prompt response of the health shocks [64]	Strong leadership, tight bonds and sense of kinship at the community level and trusted communication channels to address health shocks [70] Promoting and strengthening the political momentum towards UHC facilitates its realization [41] Health sector governance will require new partnerships and opportunities for dialogue [69] The global health governance of the COVID-19 response strengthens to combat the conditions of the pandemic [37]
Accountability	Effective stewardship role of the government to ensure the progress towards UHC [71] Ensuring accountability, managing health resources, and decision-making were the factors for HSG to achieve an effective and equitable health system [72]	Corruption, fear of reprisal and limited funding [73] Policy-distorting corruption can potentially prevent society from achieving health development goals [74]	Effective governance processes build strong partnerships for health and create accountability to respond to health emergencies [16]		Rule of law, engaging partners in public policy and transparency to ensure accountability [76] Integration of anti-corruption, transparency and accountability measures into health systems helps to achieve SDGs [77] Strong and transparent monitoring systems at different levels of the healthcare system can ensure accountability [78] Low socioeconomic status was the challenge to receiving healthcare services in Chile. Copayment levels fixed by law and maintaining free care for indigent and low-income families after the Universal Access with Explicit Guarantees (AUGE) programme in Chile were helpful to receive equitable and responsive health services utilization and to ensure accountability [75]

### Policy formulation and strategic plan

Of the total reviewed articles, 20 articles described their findings on policy and strategic plans. Public financing, prepaid health spending, and financial risk pooling are the successes of the health system through protecting people from economic catastrophe and helping to progress towards UHC [23, 24]. For instance, the single-payer scheme after the novel UHC system introduced in Indonesia is an example of improving health equity and access through accommodating diversity with flexible and adaptive implementation features and quick evidence-driven decisions [25]. Expansion of pro-poor healthcare services in Sri Lanka also resulted in good progress towards UHC [26]. Decentralization of healthcare services was another health system success that impacted health equity, efficiency and resilience [27]. For example, village health volunteers in Thailand, lady health workers in Pakistan and health extension workers (HEWs) in Ethiopia are successful community-based models that have contributed immensely towards health programmes [28]. On the contrary, shortages of human resources and medical supplies; poor physical healthcare access; disparities in health service utilization; and sociocultural barriers of users, such as lack of education/information and decision-making autonomy [29–31], were the challenges for the progress towards UHC. In addition, socially excluded population groups received health services from a dysfunctional publicly provided health system marked by gaps and often invisible barriers in Guatemala and Peru, which undermines the progress towards UHC [32].

Refresher or other types of training, supervision, clear policies on reward systems and good management support helped community health workers to give good quality of care in Botswana [33], and integration of human resource planning with health emergency planning during the cholera epidemic [34] were successful interventions to control health emergencies and epidemics. However, a centralized one-size-fits-all health system approach [25] and unclear administrative roles and command structures during health emergencies [16] were among the challenges to building a better future and realizing health security. For instance, Indonesia’s one-size-fits-all approach was not successful since it did not address the complexity and diversity of people [25]. Absence of clear judicial, executive and legislative authority and clarity of structures in conflict settings in Syria [35] were additional challenges to combatting the crisis.

Resilient health workforces at country levels were critical to ensuring health security and support planning and health prioritization [36]. Moving away from a one-size-fits-all health system approach in guiding pandemic response, political commitment, fair contribution and distribution of resources were the strategies that sped up the path towards UH [37, 38]. An economic architecture, rebuilding HSG and conceptualizing health workforce governance had progressive changes on health service delivery. It helped reduce poverty, unemployment and inequities [35, 39, 40], facilitating UHC and health security. Effective strategic planning, regulation, management of the health workforce, and well-designed community-driven initiatives were the strategies to combat health shocks and achieve UHC and health security [30, 41].

### Intelligence

In our review, 12 articles reported their findings on generating health system intelligence towards UHC and health security. Countries that have systems/setups on how to use intelligence or health technology assessments were helpful to inform policy and decision-making [42]. The adoption of high-quality HIS to monitor effective global and national health inequalities [43] and community-based household registers for health [44] succeeded in improving health system outcomes and facilitating the realization of UHC. Mobile phones and the internet also create an opportunity to improve access to appropriate knowledge and advice to realize UHC [45]. Innovative data management in a unified process assisted in providing timely response for patient care outcomes in Ethiopia [46]. On the other hand, the neglect of effective people-centred healthcare information was a challenge in making essential health service accessible to users [47].

An independent, objective and transparent assessment of health system gaps is needed to ensure early detection, prevention and response to biological threats [48]. Strong surveillance capacity and investments help to improve the health system's strength during a crisis. A timely response is needed to meet the national and global health goals [16]. However, late responses due to inadequate surveillance and lack of routine data combinations weakened the functionality of plans [49]. Strategic direction to sustain the achievements of a digital data culture and an automated single reporting system for multiple stakeholders are necessary to make the system user-friendly [50]. Strengthening local-level managers' ability is critical leverage in supporting informed local decision-making [51]. Using indicators to monitor health information assessment in the private sector and subnational levels helps ensure data quality and response early to public health threats [52].

### Regulation

Ten articles described the successes and challenges of legal and regulatory instruments in governing health systems to realize UHC and global health security (GHS). Establishing a national and subnational regulatory agency was crucial to monitor and enforce laws and regulations to access emergency care without regard to the ability to pay [53]. For instance, a centralized public regulatory authority of the redistributive funding model in French successfully reduced financial barriers to access health services for the poor population [54]. Mentorship and enhanced supervision of health staff also improved the quality of care at health facilities [44]. In addition, regulators encouraged investing in gauging their performance and information sharing [53], and regulating the cost of private healthcare facilitated UHC progress [55]. For example, close monitoring at all levels on the trends of key indicators and taking early corrective measures brought good health returns/outcomes in Rwanda [56]. On the other hand, lack of enforcement was an obstacle to providing free healthcare services to the poor in government-subsidized private hospitals in Delhi [57]. Political interference and unclear roles and responsibilities of different governmental regulatory bodies contributed to failures in health service delivery [55], and inadequacies in the human resource capacities of the regulatory organizations [57] were also significant challenges that affected the path towards UHC. For instance, the regulatory architecture for healthcare in Mongolia resulted in poor affordability and quality of private care [55].

Adequate training and supportive supervision of community health workers (CHWs) are helpful for saving the lives of patients during emergencies [58]. Adopting strategic purchasing and ensuring an independent accreditation system/organization accelerates progress towards better healthcare quality [27]. International treaties, constitutional and statutory law, regulations, guidelines, protocols and informal practice patterns are instrumental to govern the health system and improve service delivery [59]. For instance, applying the regulation in medical and pharmaceutical practices in the healthcare system helps improve supply chain management [60].

### Collaboration and coalition

Out of the total review articles, 14 articles’ findings presented collaboration and coalition. Coordination between public, private for-profit and not-for-profit sectors was needed to optimize the health service delivery [19]. Strong partnership with national and global actors is required to avoid late responsiveness of the health system [61]. A holistic and integrated health service delivery helped in the prevention of resource fragmentation and improved efficiency in healthcare delivery [62]. For example, coordination with nongovernmental organizations (NGOs) and local councils in conflict settings in Syria was helpful for addressing health system fragmentation [35]. Participatory governance platforms, such as the national health assembly in Thailand, are another crucial pillar for countries seeking to achieve UHC [63]. On the other hand, poor leadership practices at the subnational and national levels were the main challenges, which led to poor coordination and the absence of a prompt response to particular health shocks [64].

Multisectoral collaboration within and across sectors improves international health regulations [65] and helps maintain national health security. For example, the Port of Spain Declaration for the prevention of noncommunicable diseases (NCDs) in the Caribbean region [66] and the Global Health Security Agenda (GHSA) to maintain a safe and secure world from infectious disease threats [67] were a successful outcome of multisectoral negotiations and health diplomacy efforts. In addition, collective action by all key stakeholders through a multipronged approach also assisted in mitigating, preventing and fighting health security threats [68].

Health sector governance will require new partnerships and opportunities for dialogue between state and non-state actors [69]. Strong leadership, tight bonds, a sense of kinship at the community level and trusted communication channels across the key actors in the health system are helpful to address health shocks [70]. Promoting and strengthening the political momentum towards UHC with the involvement of all stakeholders facilitates its realization [41]. For instance, the global health governance of the COVID-19 response might be seen as international cooperation to combat pandemic conditions [37].

### Accountability

Of the total articles reviewed, the findings of nine articles described accountability. The influential stewardship role of the government accelerated the progress towards UHC [71]. Ensuring accountability, managing health resources, and decision-making were significant factors for HSG in realizing effective and equitable health services [72]. However, corruption, fear of reprisal and limited funding were major challenges in implementing effective social accountability interventions [73] and realizing UHC. Policy-distorting corruption can potentially prevent the achievement of health development goals [74]. Low-socioeconomic-status service users were another challenge to equitable provision of healthcare services. Copayment levels fixed by law, maintaining free care for indigent and low-income families in the national health fund (FONASA [*Fondo Nacional de Salud*]) after the introduction of Chile’s Universal Access with Explicit Guarantees (AUGE) programme, were helpful in promoting equitable and responsive health services and ensuring accountability [75] (Additional file 3).

Effective healthcare governance builds strong health partnerships and creates accountability mechanisms for responding to health emergencies [16]. Strengthening the rule of law, engaging partners in public policy and maintaining transparency are important to ensure accountability in the health system [76]. Integration of anti-corruption, transparency and accountability measures into health systems strengthening also supports the achievement of SDGs [77]. Transparent and robust monitoring systems in different healthcare systems are also key strategies to ensure accountability [78] (Additional file 4).

## Discussion

We explored the successes and challenges of HSG towards UHC and health security in the context of access, equity, quality, responsiveness, safety, efficiency, sustainability, financial risk protection and performance/coverage. HSG is the main change-maker for UHC and health security at the national and international levels, and is fundamental for improving the efficiency, resilience and responsiveness of the health system [15]. Decentralization of health services, the support of stakeholders, strong political and institutional structures, fair contribution, and distribution of resources through appropriate health financing modalities were the main successes of HSG in facilitating UHC and health security [27, 38, 79]. On the contrary, limited execution of similar approaches to all and ineffective people-centred healthcare information were challenges in guiding the pandemic response and access to essential health services [25, 47]. Corruption and poor coordination were also challenges that hindered progress towards UHC and health security [64, 73].

The global political momentum towards UHC and health security also provides a welcome opportunity to scale up efforts to dismantle barriers to accessing comprehensive essential health services in the pandemic context [79]. The adoption of pro-UHC policies and strategic plans, decentralization of health services, and fair contribution and distribution of resources by appropriate health financing modalities demonstrated a desire to progress towards UHC [27, 38, 76] and health security. Such pro-UHC policies and strategic plans are ultimately aimed at expanding financial coverage of and access to equitable and quality healthcare services to maintain health security. Optimizing context-specific strategic health financing modalities was a crucial intervention for access to fair healthcare services for all. For example, the single-payer scheme in Indonesia [25], pro-poor-orientated healthcare funding in Sri Lanka [26] and a combined health financing scheme in Thailand [80] were successful strategies to realize UHC. Myanmar's three-phase National Health Plan (NHP) facilitates realizing UHC by 2030 through increased access, equity and financial protection [81]. Vietnam’s Master Plan (VMP) for UHC by 2020 [82] and China’s 2009 Health Reform Plan (HRP) to establish UHC [83] were among the successful strategic plans.

On the contrary, executing the same health governance approach to all in guiding pandemic preparedness and response in Indonesia [25] was among the major challenges to delivering essential healthcare services. This approach may not address the complexity and diversity in population density and dispersion across islands, diet, diseases, local living styles and community participation. Therefore, studies indicated that moving away from a one-size-fits-all approach in guiding pandemic response [37] helped accelerate the path towards UHC and health security.

The adoption of robust HIS, including data collection, analysis, interpretation and reporting practices across health systems, is needed to enable effective global and national health inequality monitoring to support the equity-oriented realization of UHC and to meet health security-related goals during pandemics/health crises [16, 43]. An independent, objective and transparent assessment of health system gaps is needed to ensure early detection, prevention and response to biological threats [48]. For example, quality data management in a unified process assists in providing a timely response for patient care outcomes in Ethiopia [46]. On the other hand, the neglect of adequate people-centred healthcare information was a challenge in making essential health service accessible to users [47]. A strategic direction is needed to sustain the achievement of a digital data culture and an automated single reporting system for multiple stakeholders to make the system user-friendly [50]. A comprehensive and integrated disease surveillance approach assisted in generating information to minimize inefficiencies in disease-specific surveillance silos and improved health security for informed decision-making [84].

International treaties, constitutional and statutory law, regulations, guidelines, protocols and informal practice patterns are instrumental in governing health systems [59]. Establishing a national and subnational regulatory agency with sufficient authority to monitor and enforce laws and regulations was crucial to enabling access to emergency care without regard for ability to pay [53] in order to achieve UHC and health security. Cultivating bottom-up and top-down forms of accountability can strengthen the HSG to influence the quality and coverage of health services [85]. A top-down administrative approach helped to implement policy documentation/direction and was the most common measure for policy enforcement [86]. Promoting UHC for socially excluded populations helped to establish participatory spaces for policy dialogue and ensure accountability within the health systems [32]. Regulation of funds management also emphasized the need for local accountability—for instance, the management of funds in an appointed account, where the community credit cooperative was responsible for supervising the use of the fund by village clinics in a cooperative medical scheme in Masheng County, China [87].

Collaboration and integration of pandemic planning across sectors and jurisdictions would result in better preparedness [88]. UHC also provides a welcome and unifying platform for the global health community [79] and guides health systems implementation [89]. Complex political and institutional issues were found to influence UHC [90], becoming a political priority [13]. Poor leadership practices that led to poor coordination at the subnational and national levels were challenges in responding to health shocks [64]. This indicated that the public sector alone cannot achieve UHC and health security without the participation of all key stakeholders, including the private sector and the communities. This collective action can help nations in preventing and combatting health security threats [68]. In addition, this can help countries avoid resource fragmentation and improve efficiency [62]. For example, the Port of Spain Declaration in the Caribbean region and GHSA [66, 67] contribute to the UHC journey by increasing health service coverage and improving efficiency.

Governance can be strengthened through improvement in either a bottom-up form of accountability between clients and providers or a top-down form of accountability by holding policy-makers more accountable for services, and by making policy-makers better positioned to influence the quality and coverage of services [85]. The bottom-up form of accountability might be accomplished by tailoring services to the specific needs of local users, becoming effective monitors of providers, and improving choice and participation. The top-down approach can also be achieved by making information more accessible and improving supply-side functions [91]. However, health system fragility and bottlenecks, including corruption, fear of reprisal and limited funding, were major constraints to achieving global health initiatives [73]. In the absence of adequate social accountability, service providers, NGOs and civil society organizations (CSOs) sought to hold the government accountable for limitations in the health system. For instance, service providers introduced structures to increase accountability at the service level and their interactions with the higher health system levels in response to poor government-led healthcare facility governance and service inefficiencies in Malawi [72]. Citizen-led accountability initiatives in Guatemala and Peru also revealed serious deficiencies that undermined efforts to realize UHC in both countries. In these countries, social exclusion remains embedded and undermines the health coverage provided to marginalized populations [32]. Donors, governments and other actors promote UHC for socially excluded populations, which helps to democratize and strengthen the participatory spaces for policy dialogue and accountability processes within their health systems.

**Fig. 2:**
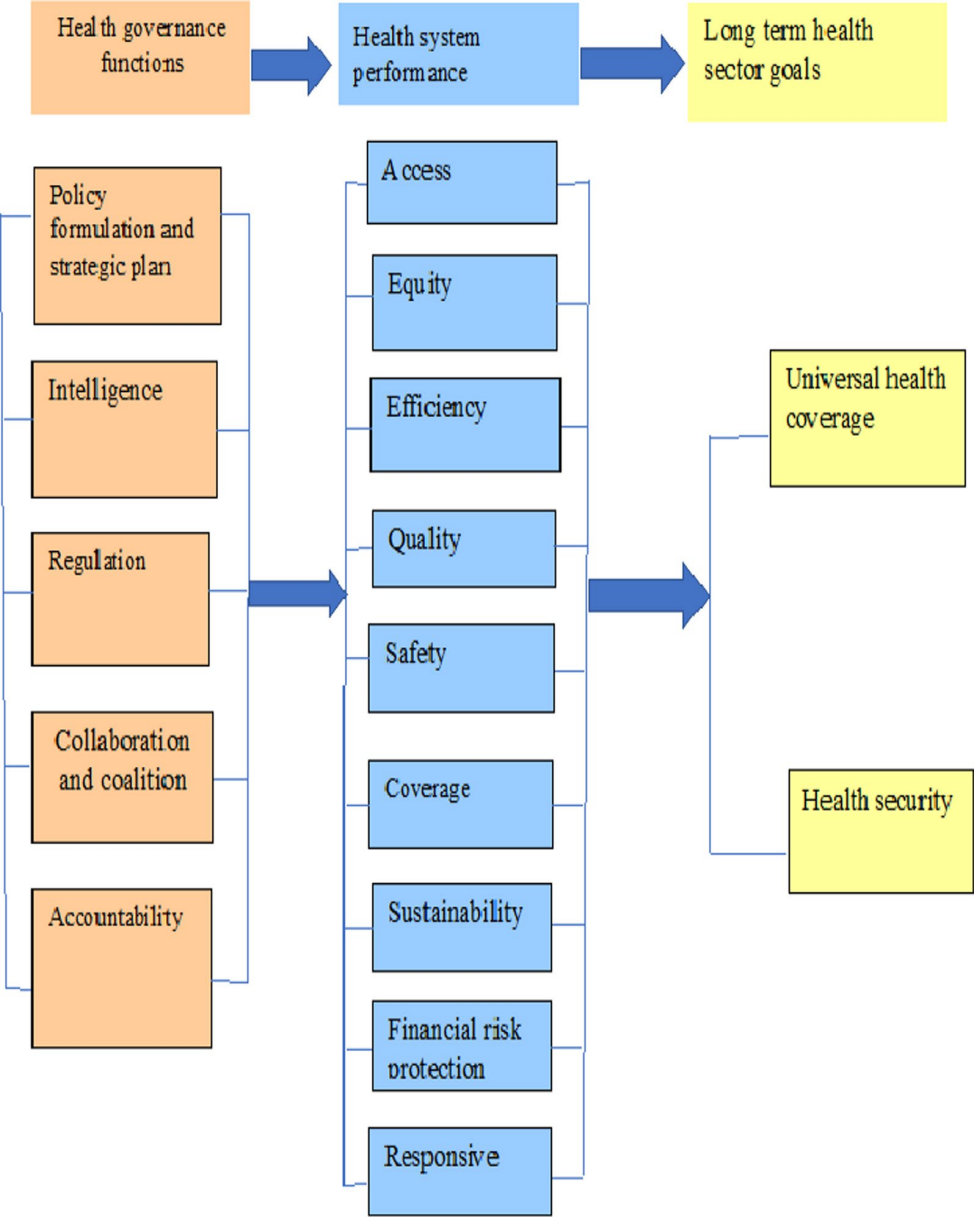
Relationship between HSG, and UHC and health security, 2021

Strong HSG can result in high health system performance, including access, equity, quality, coverage, safety, efficiency, sustainability, responsiveness and financial risk protection. In turn, high-quality health system performance can help to achieve the long-term health sector goals, including UHC and health security. In this review, we summarized the impact and interrelationship of HSG with respect to the health sector SDGs, including UHC and health security (Fig. 2).

### Implications

This review has several policy implications, since HSG underpins the other health system building blocks. First, this review provides insight into the impact of HSG on the delivery of basic essential health services to facilitate progress towards UHC and its effect on reducing health security-related threats during the COVID-19 pandemic era. Second, it raises awareness for health programmers and policy-makers on the importance of HSG as a key function of the health system and provides a path for discussion on various subjects. Third, it also provides insight into key HSG functions, such as formulating policy and strategic planning, design regulation, generating intelligence, building collaboration, and ensuring accountability for improving these practices by developing interventions instituted at the policy or implementation level of the health system. Fourth, the findings of this review could help policy-makers and government officials to revise and update their financial and other plans. Fifth, this review could assist policy-makers in preparing a global-level stewardship-based set of indicators to monitor the HSG function at various levels. Sixth, this review also provides insight for policy-makers to introduce a strong accountability system within public institutions to provide more inclusive and equitable health services without excluding any population groups, and thus facilitate progress towards UHC. Finally, this review will help future researchers as baseline information for those interested in conducting related studies. The findings will also help in designing interventions to address challenges, scale up successes, and implement different initiatives and/or projects.

### Study limitations

A comprehensive review could provide evidence of HSG on UHC in the pandemic context. This study was a mixed-studies review and included all kinds of studies (quantitative, qualitative and mixed methods). In such a review, the contexts and purposes emphasize the ability of the studies to answer our review questions rather than the types and quality of the primary studies themselves. In this review, we used the same processes and followed some components of the PRISMA checklist, but missed meta-analysis components. In addition, we used the JBI critical appraisal checklist to specify some methodological criteria for academic literature, but we did not do a quality appraisal for grey literature, and we did not exclude articles using quality appraisal assessment of the findings alone. Thus, we did not use the grading of findings and recommendations of the included studies.

## Conclusion

Strong HSG is required to accelerate progress towards UHC and health security. UHC and health security will be achieved by adopting a strategic policy and plan, generating a robust health system intelligence, advocating intersectoral collaboration, regulating health stakeholders and ensuring accountability. Decentralization of health service to the grassroots level, support of stakeholders, fair contribution and distribution of resources by appropriate health financing modalities were also successful HSG strategies in advancing towards UHC during health crises. On the other hand, the execution of similar HSG approaches to all for guiding pandemic response, government policies and global health diplomacy were challenges that affected the path towards UHC and health security. Strengthening the political commitment, such as financing UHC during pandemics, will facilitate progress towards UHC and health security. Optimizing context-specific strategic health financing modalities will also be essential for providing users with quality and equitable health services. Integrating independent regulatory accreditation organizations within the health system, and quality and equity healthcare service indicators into the national social protection monitoring and evaluation system will speed up the progress towards UHC and health security. Further research should explore new opportunities and challenges of HSG towards UHC and health security to improve healthcare delivery, particularly in specific geographical locations/nations.

## Supplementary Information

**Additional file 1**: Search strategy.

**Additional file 2**: ENTERQ checklist.

**Additional file 3**: Characteristics of included articles.

**Additional file 4**: Focuses and contexts of key findings.

## Data Availability

Not applicable.

## Abbreviations

ENTREQ: Enhancing Transparency in Reporting the Synthesis of Qualitative Research
GHS: Global health security
GHSA: Global Health Security Agenda
HSG: Health systems governance
JBI: Joanna Briggs Institute
MDGs: Millennium Development Goals
NCDs: Noncommunicable diseases
NGOs: Nongovernmental organizations
UHC: Universal health coverage
SDGs: Sustainable Development Goals
