# Supplementary material for: Successes and challenges of health systems governance towards universal health coverage and global health security: a narrative review and synthesis of the literature
Source: Health Res Policy Syst. 2022 May 2;20:50. doi: 10.1186/s12961-022-00858-7 (PMC9059443; doi:10.1186/s12961-022-00858-7)
Supplement: Supplementary file 1 — Additional file 1: Search strategy. [file 12961_2022_858_MOESM1_ESM.docx]

**Search strategy**

- ((Health system*[Title/Abstract] OR Health Service*[Title/Abstract]) AND (Resilien*[Title/Abstract] OR strong [Title/Abstract] OR robust [Title/Abstract] OR health security [Title/Abstract] OR universal health coverage [Title/Abstract])) AND (Governance [Title/Abstract] OR Strategy plan [Title/Abstract] OR Policy formulation [Title/Abstract] OR intelligence[Title/Abstract] OR information[Title/Abstract] OR regulation[Title/Abstract] OR accountability[Title/Abstract] OR steward*[Title/Abstract]))
