# Supplementary material for: Successes and challenges of health systems governance towards universal health coverage and global health security: a narrative review and synthesis of the literature
Source: Health Res Policy Syst. 2022 May 2;20:50. doi: 10.1186/s12961-022-00858-7 (PMC9059443; doi:10.1186/s12961-022-00858-7)
Supplement: Supplementary file 3 — Additional file 3: Characteristics of included articles. [file 12961_2022_858_MOESM3_ESM.docx]

Table 3: Characteristics of included articles, 2021

| S.no | Author and year | Title of article | Settings | Type of article | Design | Participants | Data sources | Analysis/ | Major-topic/ outcome discussed |
| --- | --- | --- | --- | --- | --- | --- | --- | --- | --- |
| 1 | WHO,2016 | Universal Health Coverage: Moving Towards Better Health Action Framework for the Western Pacific Region | Global | WHO |  |  | - Articles - Experts’ opinion | Narrative | Priority actions for improving accountability include strengthening government leadership and rule of law for health, engaging in partnerships for public policy, and ensuring mechanisms for transparent monitoring and evaluation. |
| 2 | Okoronkwo et al., 2014 | The long walk to universal health coverage: patterns of inequities in the use of primary healthcare services in Enugu, Southeast Nigeria. | Nigeria | Quantitative | Cross-sectional | - Adult service users | Interviews | Chi-square | Utilization of PHC services in Nigeria varies across educational level, residence, gender and socio-economic status of the service users. |
| 3 | van Olmen et al., 2010 | Analysing health systems to make them stronger. | Global | Books |  |  | - Articles - Experts’ opinion | Narrative | State actors, at both central and peripheral levels, need to take up leadership of coordination mechanisms to optimise the health service delivery through coordination between public, private for-profit and not-for-profit sectors. |
| 4 | Eyawo et al, 2020 | Rethinking the central role of equity in the Global Governance of pandemic response | Global | Perspective |  |  | - Experts’ opinion - Articles | Narrative | Global health governance needs to move further away from the advocacy of a one-size-fits-all approach in guiding pandemic preparedness and response strategies. The global health governance of COVID-19 response strengthens to combat the conditions of the pandemic. |
| 5 | Rosewell et al, 2013 | Human resources for health: lessons from the cholera outbreak in Papua New Guinea. | Papua New Guinea | Lessons from the Field |  |  | - Experts’ opinion | Narrative | Integration of human resource planning with health emergency planning to maintain and strengthen the experiences gained during cholera outbreak. |
| 6 | Scott et al, 2017 | Exploring how different modes of governance act across health system levels to influence primary healthcare facility managers’ use of information in decision-making: experience from Cape Town, South Africa. | South  Africa | Qualitative | Multiple case  study | - policy and guidelines - Facility managers | - Document review - Interview - Observation | Explanatory | Strengthening local level managers’ ability to create enabling environments is an important leverage point in supporting informed local decision-making. |
| 7 | Harrington et al, 2013 | Detecting and responding to a dengue outbreak: evaluation of existing strategies in country outbreak response planning. Journal of tropical medicine. | Global | Review |  |  | Articles | Framework analysis | Late timing of responses due to poor surveillance, lack of combining routine data with additional alerts, and lack of triggers for initiating the response weakened the functionality of plans. |
| 8 | Chattu et al, 2020 | Canada’s role in strengthening global health security during the COVID-19 pandemic. | Canada | Commentary |  |  | - Articles - Experts’ opinion | Narrative | Collective action by all key stakeholders through a multi-pronged approach to mitigate, prevent and fight against health security threats (now and in future) through global health diplomacy |
| 9 | Karamagi et al., 2021 | Towards universal health coverage in the WHO African Region: assessing health system functionality, incorporating lessons from COVID-19 | Africa | Quantitative |  |  | - Publicly available sources | Correlation | Public financing provides the biggest improvement  in health system functioning compared with external and out-of-pocket financing. |
| 10 | Shoman et al, 2017 | The link between the West African Ebola outbreak and health systems in Guinea, Liberia and Sierra Leone: a systematic review. | Guinea, Liberia and Sierra Leone | Review |  |  | - Articles - Expert interviews | Framework analysis | Poor leadership at the national government level was the main reason that led to the poor coordination and absence of a prompt response. |
| 11 | Alonge O,2019 | Understanding the role of community resilience in addressing the Ebola virus disease epidemic in Liberia: a qualitative study (community resilience in Liberia). | Liberia | Qualitative | Qualitative study design | Community members | Key informant interviews | Community resilience model | Strong leadership, tight bonds and sense of kinship at the community level; trusted communication channels; and trust among various key actors in the health system are needed to address health shocks. |
| 12 | Ayanore et al, 2019 | Towards Resilient Health Systems in Sub-Saharan Africa: A Systematic Review of the English Language Literature on Health Workforce, Surveillance, and Health Governance Issues for Health Systems Strengthening. | Sub-Saharan Africa | Review |  |  | - Articles | Narrative | Effective governance processes build strong partnerships for health and create accountability to respond the health emergencies. The absence of clear administrative roles and command structures can lead to high health workforce attrition rates, particularly in health emergencies. Timely response needed to meet the national and global health goals. Surveillance capacity and strong investments to improve the strength of the health system during crisis. |
| 13 | Walt et al, 1989 | Community health workers in national programmes: the case of the family welfare educators of Botswana | Botswana | Mixed | Case study | - Documents - Policy makers - Family welfare educators. | - Document review - Interview | Narrative | Refresher or other types of On-the-job training, supervision, clear policies on reward systems and good management support helped for community health workers to give good quality of care during emergency health threats in Botswana. |
| 14 | Assan, et al. 2019 | Challenges to achieving universal health coverage through community-based health planning and services delivery approach: a qualitative study in Ghana | Ghana | Qualitative | Qualitative study design | National, regional, district and local levels managers | - Interviews (in-depth) | Thematic analysis | UHC is a political choice and needs a coordinated involvement of all stakeholders, from community members to international partners to realize it. Well-designed social mobilization and community-driven initiatives have proved capable to establish a more substantial means at the center of health policy planning and implementation to achieve UHC. |
| 15 | Bhutta et al., 2010 | Global experience of community health workers for delivery of health-related millennium development goals: a systematic review, country case studies, and recommendations for integration into national health systems. | Global | Review | Case study |  | Articles | Narrative | Village health volunteers in Thailand, the lady health workers in Pakistan, the health extension workers in Ethiopia and building resources across communities in Bangladesh are all successful community-based models contributed immensely towards health programmes. |
| 16 | WHO, 2010 | Monitoring the building blocks of health systems: A handbook of indicators and their measurement strategies | Global | WHO |  |  | - Articles - Experts’ opinion | Narrative | Resilient in health workforces at country levels were critical to ensuring health security and support planning and health prioritisation. |
| 17 | Drobac et al. 2013 | Comprehensive and integrated district health systems strengthening: The Rwanda population Health Implementation and Training (PHIT) Partnership | Rwanda | Study Protocol | PHIT interventional design | - Households - Documents | - Interview - Document review | Difference-in-Differences | Mentorship and enhanced supervision of health staff improves quality care at health facilities. The use of community health household registers improves health system outcomes. |
| 18 | Cho et al., 2015 | Two epidemics and Global health security agenda | Global | Editorial |  |  | - Articles - Experts’ opinion | Narrative | An independent, objective and transparent assessment of health system gaps at all levels for ensuring early detection, prevention, and rapid response to biological threats. |
| 19 | Sayinzoga et al, 2016 | Drivers of improved health sector performance in Rwanda: A qualitative view from within. | Rwanda | Qualitative | Web-based survey | Directors of health | - Self-ad mistered | Narrative | Close monitoring at all levels of trends in key indicators and early corrective measures distinguish Rwanda’s health system from other countries and brought good health outcomes. |
| 20 | Wong et al., 2009 | Developing patient registration and medical records management system in Ethiopia. | Ethiopia | Quantitative | Pre-post | - Physicians - Records | - Interview - Record review | t-test | Novel data managing processes, such as merging patient registrations and medical records into a unified process assists in providing a timely response for patient care outcomes in Ethiopia. |
| 21 | Sherr et al., 2013 | Strengthening integrated primary health care in Sofala, Mozambique. | Mozambique | Study Protocol | Quasi-experimental | - Households - Service users | - Demographic health/ multi-indicator cluster survey | Difference-in-Difference | A holistic and integrated services can avoid resource fragmentation and improve efficiency in health care delivery. |
| 22 | Coovadia et al., 2009 | The health and health system of South Africa: Historical roots of current public health challenges. | South Africa | Series |  |  | - Articles - Experts’ opinion | Narrative | An economic architecture that allows the development of programmes that reduce poverty, unemployment, and inequities are essential for health systems strengthening |
| 23 | Gostin et al, 2015 | A retrospective and prospective analysis of the West African Ebola virus disease epidemic: Robust national health systems at the foundation and an empowered WHO at the apex. | West Africa | Public policy |  |  | - Articles - Experts’ opinion | Narrative | At the apex of global health governance, strong partnership with the WHO and national and global actors is required to avoid late-responsiveness, such as during the Ebola crises |
| 24 | Siekmans et al. 2017 | Community-based health care is an essential component of a resilient health system: Evidence from Ebola outbreak in Liberia | Liberia | Mixed | Mixed | Health workers and project staffs | - Survey - Interviews (FGDs) | Content, and  Fisher’s exact test | In critical emergencies, adequate training and guidelines with supportive supervision to community health workers help deliver lifesaving services to patients |
| 25 | Van Olmen, 2011 | The growing caseload of chronic life-long conditions calls for a move towards full self-management in low-income countries. | LICs | Debate |  |  | - Articles - Experts’ opinion | Narrative | Rapid increases in connectivity to mobile phones and the Internet are creating opportunities for improving access to appropriate knowledge and advice which contribute to the achievement of UHC depends, to a considerable extent, on government action. |
| 26 | Reynolds, 2017 | Strengthening health systems to provide emergency care. | Global | Book chapter |  |  | - Articles - Experts’ opinion | Narrative | Establishment of a national and sub-national regulatory agency with sufficient authority to monitor and enforce laws and regulations that protect access to emergency care. |
| 27 | Chattu et al, 2018 | Port-of-Spain Declaration for global NCD prevention. | Caribbean countries | Correspondence |  |  | Articles | Narrative | The chronic non-communicable diseases (NCDs) pose a threat to the health security of the nations, regions and now became a global epidemic. The high-level commitment, lengthy persistent negotiations and successful health diplomacy efforts in the Caribbean region over a decade resulted in “Port of Spain Declaration” in 2007. |
| 28 | Gottret et al, 2008 | Good practices in health financing: Lessons for reforms in low and middle-income countries. | LMICs | Book |  |  | Articles | Narrative | Sri Lanka has achieved near universal health coverage and has done so with remarkably low health expenditure through the expansion of health care coverage with pro-poor orientation of health system that is free at the point of delivery to all citizens. |
| 29 | Abimbola et al., 2019 | The impacts of decentralization on health system equity, efficiency and resilience: a realist synthesis of the evidence. | Global | Review |  |  | Articles | Theoretical framework | Decentralization of healthcare services impacts on health system equity, efficiency and resilience. |
| 30 | Ranabhat et al.,2019 | Challenges and opportunities towards the road of UHC in Nepal: a systematic review. | Nepal | Review |  |  | Articles | Narrative | Political commitment, fair contribution and distribution of resources by appropriate health financing modality can speed up the path of UHC. |
| 31 | Khan et al, 2009 | Anti-corruption in adverse contexts: strategies for improving implementation. | LICs | Working paper |  |  | Articles | Narrative | Policy-distorting corruption can potentially prevent from achieving health development goals. |
| 32 | Douedari et al, 2018 | Perspectives on Rebuilding Health System Governance in Opposition-Controlled Syria: A Qualitative Study | Syria | Qualitative | Qualitative study design | Health directorates, non-governmental organisations, donors, and service-users | In-depth interview | Thematic | In Syria, no clear judicial, executive, legislative authority, and clarity of structures, but rebuilding HSG through supporting health directorates had progressive changes on health service delivery in opposition-controlled areas. Coordination with other actors, such as health directorates, NGOs, and local councils could help to address health system fragmentation and competition in the absence of a united authority. |
| 33 | Dube-Mwedzi et al, 2020 | A rapid assessment of the National Regulatory Systems for medical products in the Southern African Development Community | Southern Africa | Review |  |  | Articles | Content | The efficiency and effectiveness of regulatory systems are beginning to take a central stage, and regulators must be encouraged to invest in gauging their performance and information sharing. It shall be exercised in medical and pharmaceutical practices in the healthcare system to improve the supply chain management. |
| 34 | Koller et al, 2020 | Promoting anti-corruption, transparency and accountability to achieve universal health coverage | Global | Editorial |  |  | - Articles - Experts’ opinion | Narrative | Integration of anti-corruption, transparency and accountability measures into health systems strengthening helps to achieving SDGs. |
| 35 | Masefield et al., 2020 | Challenges to effective governance in a low-income healthcare system: a qualitative study of stakeholder perceptions in Malawi | Malawi | Qualitative | Qualitative study design | NGOs, civil society, and local government  bodies | In-depth interview | Inductive content | Ensure accountability, managing health resources and influence in decision-making are the significant factors for health governance to achieve a more effective and equitable health system. |
| 36 | Nay et al, 2016 | Achieving universal health coverage in France: policy reforms and the challenge of inequalities | France | Series |  |  | - Articles - Experts’ opinion | Narrative | The French healthcare system promotes the principle of healthcare insurance based on a redistributive funding model with a high level of institutional diversity to reduce financial barriers to access for the poor populations at a reasonable cost. |
| 37 | Pyone al, 2020 | Health system governance in strengthening International Health Regulations (IHR) compliance in Myanmar | Myanmar | Review |  |  | - Articles - Experts’ opinion | Narrative | Multisectoral, collaborative working within and across sectors is fundamental to improving IHR (2005) compliance, and for that, governance is the best lever of the health system. |
| 38 | Sivalal et al, 2009 | Health technology assessment in the Asia Pacific region. | Asia Pacific region | Perspective |  |  | - Articles - Experts’ opinion | Narrative | Countries that have systems/ set ups on how to use intelligence or health technology assessments were helpful to inform policy and decision making. |
| 39 | Begum et al, 2020 | Perceptions and experiences with district health information system software to collect and utilize health data in Bangladesh: a qualitative exploratory study. | Bangladesh | Qualitative | Grounded theory | Statisticians’ nurses, health inspectors, health-managers  , HMIS-experts, Partners, etc. | - Key informant - In-depth - FGDs | Content | Focused strategic direction is needed to sustain the achievements of digital data culture and an automated single reporting system for multiple stakeholders could make the system more user-friendly. |
| 40 | Rajan et al, 2019 | Institutionalizing participatory health governance: lessons from nine years of the National Health Assembly model in Thailand | Thailand | Review |  |  | Articles | Narrative | Improving participatory governance in health systems, for example of a participatory health governance platform, is the National Health Assembly (NHA) in Thailand is an increasingly important area and recognized as a key pillar for countries seeking to achieve UHC. |
| 41 | Royston, et al, 2020 | Universal access to essential health information: accelerating progress towards universal health coverage and other SDG health targets. | Global | Review |  |  | Articles | Narrative | Neglection of an effective people-centered healthcare information affected the access to essential health services and achieve UHC as part of SDGs |
| 42 | Uzochukwu et al., 2018 | Accountability mechanisms for implementing a health financing option: the case of the basic health care provision fund (BHCPF) in Nigeria | Nigeria | Qualitative | Qualitative study design | Policy  makers, partners and civil organizations | In-depth interviews | Thematic | Ensuring accountability encompass planning mechanisms, strong and transparent monitoring and supervision systems, and systematic reporting at different levels of the healthcare system. |
| 43 | Hosseinpoor et al, 2014 | Equity-oriented monitoring in the context of universal health coverage | Global | Review |  |  | Articles | Narrative | The development of high-quality health information systems, including data collection, analysis, interpretation, and reporting practices that are linked to review and evaluation cycles across health systems, will enable effective global and national health inequality monitoring. |
| 44 | Tumusiime et al, 2020 | Building health system resilience in the context of primary health care revitalization for attainment of UHC: proceedings from the Fifth Health Sector Directors’ Policy and Planning Meeting for the WHO African Region | Africa | Meeting report |  | WHO country representative | Meeting discussants | Narrative | Health sector governance will require new partnerships and opportunities for dialogue between state and non-state actors. |
| 45 | Agustina et al, 2019 | Universal health coverage in Indonesia: concept, progress, and challenges. | Indonesia | Review |  |  | Articles | Narrative | The centralized one size fits all approach did not address the complexity and diversity in population density and dispersion across islands, diets, diseases, local living styles, community participation. The single-payer scheme approach after the novel UHC system introduced in 2014 also improved health equity and service access through accommodating diversity with flexible and adaptive implementation features and quick evidence-driven decisions. |
| 46 | Cometto et al, 2020 | Developing the health workforce for universal health coverage. | Global | Policy and practice |  |  | Articles | Narrative | An effective strategic planning, development, regulation, oversight and management of the health workforce at the time of health shocks is essentially required for performing the bureaucratic work, such as recruitment, transfers and retirement |
| 47 | Fantaye et al, 2019 | Universal health coverage and facilitation of equitable access to care in Africa. | Africa | Review |  |  | Articles | Narrative | Physical inaccessibility, lack of education and information, decision-making power, and gender-based autonomy were still found to  deter access to and use of, health services. |
| 48 | Burkholder et al, 2020 | Governing access to emergency care in Africa. | Africa | Review |  |  | Articles | Narrative | A variety of legal and regulatory instruments that are used in governing health systems, includes international treaties, constitutional and statutory law, regulations, guidelines, protocols and informal practice patterns |
| 49 | Chattu, et al, 2019 | The need for health diplomacy in health security operations. | Global | Perspective |  |  | - Articles - Experts’ opinion | Narrative | Multi-sectoral and multilateral efforts such as the Global Health Security Agenda (GHSA) is an effort by nations, international organizations, and civil societies to speed up the progress towards a safe and secure world from infectious disease threats, and it is also a result of successful health diplomacy to promote GHS |
| 50 | Bloom et al, 2019 | Service delivery transformation for UHC in Asia and the Pacific. | Asia and Pacific region | Perspective |  |  | - Articles - Experts’ opinion | Narrative | Governments need to play an effective stewardship role to ensure that digital health services contribute to the progress towards UHC |
| 51 | Danhoundo et al, 2018 | Improving social accountability processes in the health sector in sub-Saharan Africa: a systematic review | Sub-Saharan Africa | Review |  |  | Articles | Narrative | Health system barriers, such as corruption, fear of reprisal, and limited funding appear to be major challenges to effective social accountability interventions |
| 52 | Lim et al, 2021 | Governance in health workforce: how do we improve on the concept? A network‑based, stakeholder‑driven approach | Global | Review | Network- based, stakeholder driven approach |  | Articles | Narrative | Improving the conceptualization of health workforce governance deepens our understanding of health systems governance, enables the operationalization of governance policies that improve health workforce performance and ultimately delivers health gains on the path towards UHC, GHS and health systems strengthening. |
| 53 | Tsevelvaanchig et al, 2017 | Regulating the for‐profit private healthcare providers towards universal health coverage: A qualitative study of legal and organizational framework in Mongolia | Mongolia | Qualitative | Qualitative study design | Policy makers, regulatory agencies, providers, professionals, and patients | In‐depth/ key informant interviews | Narrative | Regulating the cost of private care is a growing interest for countries that are moving towards UHC. An increased political interference, governance issues, unclear roles, and responsibilities of different government regulatory bodies have contributed to failures in implementation of existing regulations. Regulatory architecture for healthcare in Mongolia is not optimally designed to improve affordability and quality of private care. |
| 54 | Erondu et al, 2021 | Improving National Intelligence for Public Health Preparedness: a methodological approach to finding local multi-sector indicators for health security | Global | Policy and practice |  |  | Articles | Narrative | Applying piloted indicators in private sector and subnational levels helps to ensure data quality and to respond public health threats. |
| 55 | Sheikh et al, 2013 | What explains regulatory failure? Analyzing the architecture of health care regulation in two Indian states | India | Policy and practice |  |  | - Articles - Experts’ opinion | Narrative | Inadequacies of the human resource capacities of regulatory organizations, notably shortages of inspectors, was the main emerging explanations for regulators’ inability to fulfil mandated roles. In Delhi, lack of enforcement of the policy for free provision of services to the poor in government-subsidized private hospitals, was ascribed to shortages of inspectors. |
| 56 | Frenz et al, 2014 | Achieving effective universal health coverage with equity: evidence from Chile | Chile | Quantitative | Cross sectional survey | Households | Chile national socioeconomic  characterization surveys (CASEN) | Binary logistic regression | Low socio-economic status was the challenge to access healthcare in Chile. Co-payment levels fixed by law, maintaining free care for indigent and low-income families in national health fund (FONASA) after Chile’s universal access with explicit guarantees program (AUGE) were helpful to receive equitable and responsive health services. |
| 57 | Moreno-Serra et al, 2012 | Does progress towards universal health coverage improve population health? | Global | Series |  |  | - Articles - Experts’ opinion | Narrative | Prepaid health spending and financial risk pooling is a crucial sign of progress towards UHC. |
| 58 | Samuel et al, 2020 | Social exclusion and universal health coverage: health care rights and citizen-led accountability in Guatemala and Peru. | Guatemala and Peru | Review |  |  | Articles | Narrative | In the cases of Guatemala and Peru, socially excluded population groups received health services from a dysfunctional publicly provided health system marked by gaps and often invisible barriers, which undermines the progress towards UHC. |
