# Supplementary material for: Successes and challenges of health systems governance towards universal health coverage and global health security: a narrative review and synthesis of the literature
Source: Health Res Policy Syst. 2022 May 2;20:50. doi: 10.1186/s12961-022-00858-7 (PMC9059443; doi:10.1186/s12961-022-00858-7)
Supplement: Supplementary file 4 — Additional file 4: Focuses and contexts of key findings. [file 12961_2022_858_MOESM4_ESM.docx]

Table 4: Focuses and contexts of key findings on HSG for UHC and health security, 2021

| Frameworks | No. of articles | Country | Focus | Context | Outcomes |
| --- | --- | --- | --- | --- | --- |
| Policy formulation and strategic plans | 20 | 14 LMIC and 6 global | GHS | Preparedness and response | - Resilient in health workforces at country levels were critical to ensuring health security and support planning and health prioritisation (World Health Organization, 2010). - Global health governance needs to move further away from the advocacy of a one-size-fits-all approach in guiding pandemic preparedness and response strategies (Eyawo and Viens, 2020). - Centralized one size fits all approach did not address the complexity and diversity in population density and dispersion in Indonesia (Agustina et al., 2019). |
|  |  |  |  | Political instability | - In Syria, no clear judicial, executive, legislative authority, and clarity of structures, but rebuilding HSG through supporting health directorates (HDs) had progressive changes on health service delivery in opposition-controlled areas (Douedari and Howard, 2019). |
|  |  |  |  | Performance | - Improving the conceptualization of health workforce governance enables the operationalization of governance policies to improve health workforce performance and the path towards UHC and GHS (Lim and Lin, 2021). - An effective strategic planning, development, regulation, oversight and management of the health workforce at the time of health shocks is essentially required for recruitment, transfers and retirement (Cometto et al., 2020). - Integration of human resource planning with health emergency planning to maintain and strengthen the experiences gained during cholera outbreak (Rosewell et al., 2013). - The absence of clear administrative roles and command structures can lead to high health workforce attrition rates, particularly in health emergencies (Ayanore et al., 2019). |
|  |  |  | UHC | Quality | - Refresher or other types of training, supervision, clear policies on reward systems and good management support helped community health workers to give good quality of care in Botswana (Walt et al., 1989). |
|  |  |  |  | Equity and access | - The single-payer scheme approach in Indonesia improved health equity and service access after the novel UHC system introduced in Indonesia (Agustina et al., 2019). - Shortages in human resources and medical supplies, socio-cultural barriers, physical inaccessibility, lack of education/ information, decision-making power, and gender-based autonomy found to deter access and utilization of health services (Cometto et al., 2020). - A greater reliance on prepaid health spending and financial risk pooling is regarded as a key sign of progress towards UHC (Moreno-Serra and Smith, 2012). - Political commitment, fair contribution and distribution of resources by appropriate health financing modality can speed up the path of UHC (Ranabhat et al., 2019). - In the cases of Guatemala and Peru, socially excluded population groups received health services from a dysfunctional publicly provided health system marked by gaps and often invisible barriers, which undermines the progress towards UHC (Samuel et al., 2020). |
|  |  |  |  | Equity | - An economic architecture that allows the development of programmes that reduce poverty, unemployment, and inequities are essential for health systems (Coovadia et al., 2009). - Physical access to essential services is the lowest capacity in most countries in Africa (Fantaye and Yaya, 2019). - Public financing provides an improvement in health system functioning compared with that of out-of-pocket financing to protect from financial catastrophe (Karamagi et al., 2021). - In Sri Lanka, the expansion of a pro-poor a free state-funded healthcare services orientation at the point of delivery to all citizens resulted in a good progress towards UHC (Gottret et al., 2008). - Decentralization of healthcare services impacts on health system equity, efficiency and resilience (Abimbola et al., 2019). - Utilization of PHC services in Nigeria varies across educational level, residence, gender and socio-economic status of the service users (Okoronkwo et al., 2014). |
|  |  |  | UHC and GHS | Community participation | - A well-designed and community-driven initiatives have proved capable of establishing a more substantial means need to be at the center of health policy planning and implementation to achieve UHC (Assan et al., 2019). - Village health volunteers in Thailand, the lady health workers in Pakistan, the health extension workers in Ethiopia and building resources across communities in Bangladesh are all successful community-based models contributed immensely towards health programmes (Bhutta et al., 2010). |
| Generate intelligence | 12 | 6 LMIC and 6 global | GHS | HS preparedness | - An independent, objective and transparent assessment of health system gaps needed to ensure early detection, prevention and response to biological threats (Cho and Chu, 2015). |
|  |  |  |  | Response | - Timely response needed to meet the national and global health goals (Ayanore et al., 2019). |
|  |  |  |  |  | - Late timing of responses due to poor surveillance and lack of combining routine data weakened the functionality of plans (Harrington et al., 2013). |
|  |  |  | UHC | Equity | - High-quality HIS enable to monitor effective global and national health inequality (Hosseinpoor et al., 2014). |
|  |  |  | GHS | Surveillance | - Surveillance capacity and strong investments to improve the strength of the health system during crisis (Ayanore et al., 2019). |
|  |  |  |  | Health information | - Applying piloted indicators in private sector and subnational levels helps to ensure data quality and to respond public health threats (Erondu et al., 2021). |
|  |  |  |  | Decentralization | - Strengthening local level managers’ ability is an important leverage point in supporting informed local decision-making to combat health crisis (Scott and Gilson, 2017). |
|  |  |  |  | Community | - Community health household registers improve health system outcomes (Drobac et al., 2013). |
|  |  |  | UHC | Quality data | - Innovative data management in a unified process, assists in providing a timely response for patient care outcomes in Ethiopia (Wong and Bradley, 2009). |
|  |  |  |  | Technology | - Mobile phones and internet are creating opportunities to improve access to appropriate knowledge and advice to realize UHC (Van Olmen et al., 2011). - Focused strategic direction is needed to sustain the achievements of digital data culture and an automated single reporting system for multiple stakeholders could make the system more user-friendly (Begum et al., 2020). - Countries that have systems/ set ups on how to use intelligence or health technology assessments were helpful to inform policy and decision making (Sivalal, 2009). |
|  |  |  |  | Access | - Neglection of an effective people-centered healthcare information affected the access to essential health services and achieve UHC as part of SDGs (Royston et al., 2020). |
| Design of regulation | 10 | 8 LMIC, 1 HIC and 1 global | GHS and UHC | Rules | - A variety of legal and regulatory instruments that are used in governing health systems, includes international treaties, constitutional and statutory law, regulations, guidelines, protocols and informal practice patterns (Burkholder et al., 2020). |
|  |  |  |  | Authority | - Establishing a national and sub-national regulatory agency with sufficient authority to monitor and enforce laws and regulations were crucial to access emergency care (Reynolds et al., 2017). |
|  |  |  |  | Access | - The French healthcare system promotes the principle of healthcare insurance based on a redistributive funding model with a high level of institutional diversity to reduce financial barriers to access for the poor populations at a reasonable cost (Nay et al., 2016). |
|  |  |  |  | Quality | - Mentorship and enhanced supervision of health staff improves quality care at health facilities (Drobac et al., 2013). |
|  |  |  |  | Efficiency | - Regulators encouraged to invest in gauging their performance and information sharing (Dube-Mwedzi et al., 2020). |
|  |  |  | UHC | Financial protection | - Regulating the cost of private healthcare improve the move towards UHC (Tsevelvaanchig et al., 2018). |
|  |  |  |  | Quality | - Political interference, unclear roles, and responsibilities of different government regulatory bodies contributed to failures service delivery (Tsevelvaanchig et al., 2018). - The regulatory architecture for healthcare in Mongolia is not optimally designed to improve affordability and quality of private care (Tsevelvaanchig et al., 2018). - Adoption of a strategic purchasing and ensure an independence accreditation system/ organization to accelerate progress on healthcare quality (Abimbola et al., 2019). |
|  |  |  |  | Quality and access | - Close monitoring at all levels of trends in key indicators and early corrective measures distinguish Rwanda’s from health systems in other countries and brought good health outcomes (Sayinzoga and Bijlmakers, 2016). - Inadequacies of the human resource capacities of regulatory organizations, notably shortages of inspectors, was one of the main emerging explanations for regulators’ inability to fulfil mandated roles (Sheikh et al., 2015). - In Delhi, lack of enforcement of the policy for free provision of services to the poor in government-subsidized private hospitals, was ascribed to shortages of inspectors (Sheikh et al., 2015). |
|  |  |  | GHS | Community participation | - Adequate trainings and guidelines with supportive supervision to community health workers (CHWs) are helpful to deliver lifesaving services to patients at the time of emergencies (Siekmans et al., 2017). |
| Collaboration and coalition | 14 | 9 LMIC, 2 HIC, and 3 global | UHC | Intersectoral action | - A holistic and integrated health service delivery helps to avoid resource fragmentation and improve efficiency (Sherr et al., 2013). - Coordination between public, private for-profit and not-for-profit sectors were needed to optimize the health service delivery (van Olmen et al., 2010). - Strong partnership with the WHO and other national and global actors is required to avoid late responsiveness of the health system (Gostin and Friedman, 2015). - Coordination with other actors, such as health directorates, NGOs, and local councils could help to address health system fragmentation and competition in the absence of a united authority (Douedari and Howard, 2019). |
|  |  |  | GHS | Health diplomacy | - High-level negotiations and health diplomacy efforts in the Caribbean region resulted in “Port of Spain Declaration” for the prevention of NCDs (Chattu and Sakhamuri, 2018). - Global Health Security Agenda (GHSA) speed up the progress towards a safe and secure world from infectious disease threats (Chattu and Kevany, 2019). - Collective action by all key stakeholders through a multi-pronged approach to mitigate, prevent and fight against health security threats (now and in future) through global health diplomacy (Chattu et al., 2020). - The global health governance of COVID-19 response strengthens to combat the conditions of the pandemic (Eyawo and Viens, 2020). |
|  |  |  | UHC | Efficiency | - A holistic and integrated health service delivery helps to avoid resource fragmentation and improve efficiency in healthcare delivery (Sherr et al., 2013). |
|  |  |  | GHS and  UHC | Community participation | - Strong leadership, tight bonds and sense of kinship at the community level and trusted communication channels to address health shocks (Alonge et al., 2019). - Participatory governance in health systems platform, such as the national health assembly in Thailand is a key pillar for seeking to achieve UHC (Rajan et al., 2019). |
|  |  |  | GHS | Response | - Poor leadership practices at the subnational and national levels were the main challenges, which lead to poor coordination and absence of a prompt response to a certain health shocks (Shoman et al., 2017). |
|  |  |  | UHC and GHS | Intersectoral action | - Multisectoral, collaborative working within and across sectors improve IHR (2005) (Pyone et al., 2020). - Health sector governance will require new partnerships and opportunities for dialogue between state and non-state actors (Tumusiime et al., 2020). |
|  |  |  | UHC | Political will | - UHC is a political choice and needs a coordinated involvement of all stakeholders from community members to international partners to realize it (Assan et al., 2019). |
| Ensure accountability | 9 | 6 LMIC and 3 global (both LMIC and  HIC) | UHC | Rules and transparencies | - Rule of law, engaging partners in public policy and transparencies to ensure accountability (World Health Organization, 2016). |
|  |  |  | UHC | Stewardship | - Effective stewardship role of the government to ensure the progress towards UHC (Bloom, 2019). |
|  |  |  | UHC and GHS | Transparency | - Integration of anti-corruption, transparency and accountability measures into health systems helps to achieve SDGs (Koller et al., 2020). |
|  |  |  | GHS | Partnership | - Effective governance processes build strong partnerships for health and create accountability to respond the health emergencies (Ayanore et al., 2019). |
|  |  |  | UHC | Equity | - Ensure accountability, managing health resources, and decision-making were the factors for HSG to achieve an effective and equitable health system (Masefield et al., 2020). |
|  |  |  | UHC | Transparency | - Strong and transparent monitoring systems at different levels of the healthcare system can ensure accountability (Uzochukwu et al., 2018). |
|  |  |  | UHC | Corruption | - Corruption, fear of reprisal and limited funding (Danhoundo et al., 2018). |
|  |  |  | UHC | Equity, access and quality | - Policy-distorting corruption can potentially prevent from achieving health development goals (Khan et al., 2019). - Low socio-economic status was the challenge to receive healthcare services. Co-payment levels fixed by law, maintaining free care for indigent and low-income families in national health fund (FONASA) after Chile’s universal access with explicit guarantees program (AUGE) were helpful to receive equitable and responsive health services (Frenz et al., 2014). |

*IHR: International health regulations; SDGs:* *Sustainable Development Goals; NGOs: non-governmental organisation*
